# Co-Flocculation of Mixed-Sized Colloidal Particles in Aqueous Dispersions Under a DC Electric Field

**DOI:** 10.3390/ma18010098

**Published:** 2024-12-29

**Authors:** Hiroshi Kimura

**Affiliations:** Department of Chemistry and Biomolecular Science, Faculty of Engineering, Gifu University, Gifu 501-1193, Japan; kimura.hiroshi.b1@f.gifu-u.ac.jp; Tel./Fax: +81-58-293-2622

**Keywords:** electric double layer, sedimentation velocity, zeta potential, DLVO theory

## Abstract

When an electric field is applied to or removed from colloidal particle aqueous dispersions, a reversible increase in sedimentation velocity of the colloidal particles, referred to as the Electrically Induced Rapid Separation (ERS) effect, is observed. While electrophoresis and other interfacial electrokinetic phenomena under applied electric fields are well-studied, the phenomena of particle aggregation and re-dispersion caused by the application and removal of the field remain largely unexplored despite their significance. Experiments using mixed aqueous dispersions of poly (methyl methacrylate) (PMMA) particles of different sizes revealed that applying an electric field induced the formation of co-flocs involving both large and small particles, significantly enhancing the sedimentation velocity. It was also observed that slight vibrational deformation hindered the ERS effect. Under uniform electrolyte concentration conditions, the ERS effect was found to depend on pH, with a stronger effect observed when the absolute value of the zeta potential was larger. These findings indicate that the presence of the electric double layer plays a crucial role in the manifestation of the ERS effect. The results of this study provide critical insights for the further application of the ERS effect.

## 1. Introduction

In polar liquids such as water, electric double layers form around colloidal particles. These electric double layers significantly influence the dispersion, aggregation, and sedimentation velocity of colloidal particles. Typically, the primary factor affecting the dispersion and aggregation of colloidal particles is the electrolyte concentration in the system, which has been succinctly explained using DLVO theory [1]. In aqueous colloidal dispersions contained in transparent vessels, the sedimentation of colloidal particles over time results in the observation of an interface between the water phase and the colloid-rich phase. Notably, in aqueous dispersions of monodisperse particles, a very sharp interface is formed [2]. While interfacial electrokinetic phenomena such as electrophoresis under external electric fields are well-known, the phenomena of aggregation and redispersion of colloidal particles caused by the application and removal of electric fields remain largely unexplored despite their critical importance.

The author has previously demonstrated that the sedimentation velocity of colloidal particles in aqueous dispersions can be reversibly altered by applying and removing an electric field on the order of several V/mm. This phenomenon is referred to as the Electrically Induced Rapid Separation (ERS) effect [2,3]. The ERS effect has been confirmed in aqueous dispersions of various dispersed materials, including polymethyl methacrylate (PMMA) particles, silica particles, and clay minerals such as bentonite. Consequently, it is anticipated that the ERS effect may also occur in dispersions containing a wide range of other dispersed materials with different compositions.

Guelcher et al. [4] investigated the intriguing flocculation behavior of colloidal particles under a “vertical” electric field applied to colloidal particles sedimented on a horizontal electrode surface. Using polystyrene latex particles (diameter 2.5–10 µm), they observed how particles flocculated under an electric field (0.02–0.1 V/mm DC) using optical microscopy. They proposed the following two models:

(I) Electrokinetic Model
  ▪ This model assumes that the interaction between the electric double layer on the particle surface and the external electric field generates an electroosmotic flow.
  ▪ This flow moves the particles on the electrode, drawing them together and leading to flocculation.
  ▪ The flocculation rate is proportional to the strength of the electric field and the *ζ*-potential of the particles.
(II) Polarization Model
  ▪ This model attributes flocculation to flows generated by non-uniform current densities on the electrode, which move the particles and cause flocculation.
  ▪ The flocculation rate depends on the square of the electric field strength and is independent of the *ζ*-potential of the particles.

Experimental results demonstrated that the relative velocity between particles was proportional to the strength of the electric field and the *ζ*-potential, supporting the electrokinetic model (I) as an accurate explanation for the observed flocculation behavior. Moreover, the study confirmed that depending on the combination of the *ζ*-potential polarity and the electric field direction, particles either flocculated or, conversely, separated, a phenomenon also predicted by the electrokinetic model. Interestingly, the particles did not come into complete contact during flocculation, and the flocculation and separation were attributed to convection in the dispersion medium. Additionally, Kim et al. [5] reported on the dynamics of two particles on an electrode under an alternating electric field.

As described above, while the flocculation and separation of particles on electrodes have been directly observed and the mechanisms discussed in detail, the behavior of particle ensembles dispersed in water under an electric field remains unclear. This is primarily due to several challenges: the difficulty in directly observing particles or flocs during sedimentation, the fact that the ERS effect results from interactions among multiple particles (involving flocculation), the significant influence of the “direction of the electric field,” ranging from horizontal to vertical, on the ERS effect, and the various impacts on particles or their flocs, such as electrophoresis, electroosmotic flow, sedimentation potential, and streaming potential. These complex phenomena make the mechanism challenging to decipher. It is evident that the electric double layer plays a crucial role, and the phenomenon can be viewed as one of the interfacial electrokinetic phenomena. However, a detailed discussion of the mechanism underlying the ERS effect requires further foundational experimental data.

While it is evident that particles in water form flocs under the application of an electric field, the exact shape of these flocs and the mechanism behind their formation remain unresolved issues. One notable feature of the ERS effect is that the interfaces observed during particle sedimentation under an electric field are predominantly “horizontal” [2,3,6]. Most particles in water, including the PMMA and silica particles used in this study, are negatively charged and are thus expected to move toward the anode due to electrophoresis under an electric field. For instance, the electrophoretic velocity of PMMA particles with a diameter of 5.0 µm is approximately 15 µm/s under a DC electric field of 0.5 V/mm [3]. If the electric field is applied for a duration of 1000 s, the PMMA particles would theoretically travel about 15 mm toward the anode due to electrophoresis. However, in practice, only a thin region near the cathode (within about 1 mm of the cathode surface) becomes transparent, and when viewed across the entire cell, there appears to be little evidence of electrophoretic motion. One possible explanation is that convection occurs within the dispersion due to the electric field. This convection may result from a combination of particle electrophoresis and electroosmotic flow of water, creating circulation within the cell. The existence of a very thin transparent layer near the cathode strongly suggests that a significant electrostatic repulsion is acting between the negatively charged particles and the cathode during the application of the electric field. Another noteworthy feature of the ERS effect is that when the electric field is removed, the effect disappears—i.e., the sedimentation velocity of the particles returns to its pre-field value. This indicates that under the electric field, the particles are “weakly flocculated”.

In this study, one of the objectives was to elucidate how particles of different sizes form flocs under an electric field. Relatively monodisperse polymethyl methacrylate (PMMA) particles were used to investigate single-particle systems and mixed large-and-small particle aqueous dispersions. This approach aimed to provide fundamental insights into the flocculation behavior of multiple particles under an electric field. Additionally, the ERS effect in PMMA aqueous dispersions was examined on a horizontally oscillating platform with the vertical direction as the axis of rotation to evaluate the extent to which external vibrations could disrupt the flocs. Furthermore, the effect of pH on the ERS phenomenon was studied using aqueous dispersions of monodisperse silica particles, which are highly resistant to pH variations. By varying the pH, the *ζ*-potential (the potential at the slipping plane near the particle surface) was altered. To eliminate the influence of varying electrolyte concentrations, sodium chloride solutions were employed to maintain a consistent electrolyte concentration across all dispersions. This approach aimed to clarify the role of the electric double layer in the ERS phenomenon.

## 2. Experimental

### 2.1. Sample Preparation

Poly(methyl methacrylate) (PMMA) spheres were purchased from Sekisui Plastics Co., Ltd. (Osaka, Japan). The density of the PMMA particles, *ρ*_PMMA_, was 1.2 g/cm^3^, and their sizes were 2.7 ± 0.4 µm, 5.3 ± 0.4 µm, and 8.0 ± 0.6 µm. In this paper, these sizes are denoted as the average particle diameters *d*_PMMA_ = 2.7, 5.3, and 8.0 µm, respectively. The *ζ*-potential was measured using an electrophoretic spectrophotometer (Zetasizer Nano ZS, Malvern Instruments Ltd., Malvern, UK), and all particles, regardless of their size, exhibited a *ζ*-potential of approximately –40 mV. The PMMA spheres were gently dispersed into purified water (Milli-Q Advantage A10, Millipore Co., Burlington, MA, USA) using a magnetic stirrer, with ion-exchange resins (AG501-X8 (D), Bio-Rad Lab., Inc., Hercules, CA, USA) coexisting in the solution. An exhaustive deionization treatment was performed for over three months. The particle volume fraction, *ϕ*, was controlled at 5.0 × 10^−4^. For mixed aqueous dispersions containing *d*_PMMA_ = 2.7 µm and 8.0 µm particles, dispersions with a total volume fraction (*ϕ*_total_) of 5.0 × 10^−4^ were prepared, along with those with *ϕ*_total_ = 8.0 × 10^−5^ to increase transmittance. In these mixed systems, the volume fractions of *d*_PMMA_ = 2.7 µm and *d*_PMMA_ = 8.0 µm particles were equal. To investigate the effect of rotational oscillation on the ERS effect, aqueous dispersions of PMMA particles with *d*_PMMA_ = 5.3 µm were used.

Aqueous dispersions of monodisperse silica particles (KE-P250, Nippon Shokubai Co., Ltd., Osaka, Japan) were used to examine the pH effect on the ERS effect. These silica particles are resistant to a wide range of pH conditions, and this study represents the first use of silica particles to investigate the ERS effect. The volume fraction of silica particles, *ϕ*_Si_, was unified at 1.0 × 10^−4^. The silica particle diameter, *d*_Si_, and density, *ρ*_Si_, were 2.5 µm and 1.9 g/cm^3^, respectively. The silica particles were dispersed into ultrapure water and deionized for over one month using ion-exchange resins. Silica dispersions were also prepared at the same volume fraction as PMMA dispersions (*ϕ*_Si_ = 5.0 × 10^−4^). For pH adjustment, nitric acid (0.1 mol/L) and ammonia water (28%) were used to prepare aqueous dispersions with pH values ranging from 2.4 to 11. All aqueous dispersions were unified to an electrolyte concentration, *c*, of 4.0 × 10^−3^ mol/L using sodium chloride aqueous solutions (0.1 mol/L). The *ζ*-potential of the silica particles was measured for each sample. The preparation of PMMA and silica particle dispersions and the *ζ*-potential measurements were conducted at 25 °C.

### 2.2. Transmittance Measurement

In this study, transmittance measurements were used to evaluate the sedimentation velocity of particles. It is well-known that for submicron-sized colloidal particles, the sedimentation behavior often follows Stokes’ approximation, provided there is sufficiently strong electrostatic repulsion between approaching particles. Under these conditions, the descending velocity of the interface can be regarded as the sedimentation velocity of the colloidal particles. In this study, the sedimentation velocity of colloidal particles or their flocs was calculated based on the descending velocity of the interface. A rectangular transparent cell (10 mm × 10 mm × 45 mm) was used, in which Type 304 stainless steel plates were set parallel to each other along the cell walls as electrodes. The gap between the electrodes was 9.8 mm. After injecting 3.5 mL of aqueous dispersion into the cell, the top of the cell was covered with Parafilm or a rubber cap. The dispersion was gently agitated and placed on a horizontal measurement stage. A laser beam with a wavelength of 632.8 nm and a beam diameter of 0.5 mm was introduced perpendicularly to the cell wall. The path length was 10.0 mm. The observation point was set at the center between the electrodes and at an appropriate height from the cell bottom (*H*_obs_), depending on each experimental condition. A DC electric field was applied to the dispersion using a synthesized function generator (FG110, Yokogawa Test and Measurement Co., Tokyo, Japan). The electric field was applied horizontally and perpendicularly to the direction of the laser beam. The ERS effect disappears when electrolysis occurs. The voltage at which electrolysis occurs varies greatly depending on factors such as the material and shape of the electrodes, the distance between the electrodes, and the purity of the ultrapure water. In deionized water, the flow of electric current is suppressed, and electrolysis tends to be inhibited. The electric field strength, *E*, was set at a maximum of 0.5 V/mm DC, sufficient to induce the ERS effect while suppressing electrolysis. Transmittance measurements were conducted at 25 °C.

### 2.3. Application of Horizontal Oscillation with a Vertical Rotational Axis

A cell containing the PMMA aqueous dispersion was placed at the center of the horizontal platform of a vibration generator (V100, Tsubosaka Electric Co., Ltd., Tokyo, Japan). The platform is 120 mm × 120 mm square, and the outline of the cell is drawn slightly larger than the actual for better visibility. The bottom of the cell was adhered to the horizontal platform using strong double-sided tape. Oscillation was applied for up to 30 min under various conditions of vibration frequency (*f*, up to 20 Hz) and peak-to-peak amplitude (*A*_pp_, up to 2.0 deg), both in the absence of an electric field and under an electric field (*E* = 0.5 V/mm DC). The cell is rectangular, and no uniform deformation or centrifugal force occurs within it. The dispersion in the region along the axis is unaffected by vibrations, while the regions closer to the walls are more significantly influenced by the vibrations. Immediately after the oscillation was removed, transmittance measurements of the PMMA aqueous dispersion (at the center between electrodes, *H*_obs_ = 27 mm) and visual observation of the cell were conducted. All experiments were performed at 25 °C.

## 3. Results and Discussion

### 3.1. Potential Energy Curves from DLVO Theory

The potential energy between particle surfaces was calculated to analyze the dispersion state of colloidal particles in water. According to DLVO theory [7,8], the potential energy is expressed as:

(1) $$V=V_{R}+V_{A}=\pi d\varepsilon \Psi_{0}^{2}\;\exp(-\;\frac{H}{L_{D}})-\frac{A_{H}d}{24H}$$

Here, the parameters represent: *V*, the total potential energy; *V*_R_, the electrostatic potential energy; *V*_A_, the van der Waals attractive potential energy; *d*, the particle diameter; *ε*, the dielectric constant of water; *Ψ*_0_, the surface potential of the particle; H, the surface separation; and *A*_H_, the Hamaker constant. The formula for *V*_R_ is applicable when *d* >> *L*_D_ [9]. The *A*_H_ values used were 6.3 × 10^–20^ J for PMMA particles [10] and 3.0 × 10^–20^ J for silica particles [11]. Assuming only 1:1-type electrolytes are present in the aqueous medium, the thickness of the electric double layer (*L*_D_) can be determined by the following equation [12]:

(2) $$L_{D}=\frac{1}{\kappa }=\sqrt{\frac{\varepsilon k_{B}T}{2000e^{2}cN_{A}}}$$

Here, *κ* is the Debye–Hückel parameter, *k*_B_ the Boltzmann constant, *T* the absolute temperature, e the elementary charge, *c* the electrolyte concentration, and *N*_A_ Avogadro’s number. At 25 °C, the *L*_D_ (m) for *c* (mol/L) can be approximated as:

(3) $$L_{D}=\frac{3.04}{\sqrt{c}}\times 10^{-10}$$

Assuming *c* = 1.0 × 10^–5^ mol/L, *L*_D_ is calculated as 96 nm. The value of *c* was estimated based on the electrical conductivity of the water used [13].

**Figure 1:**
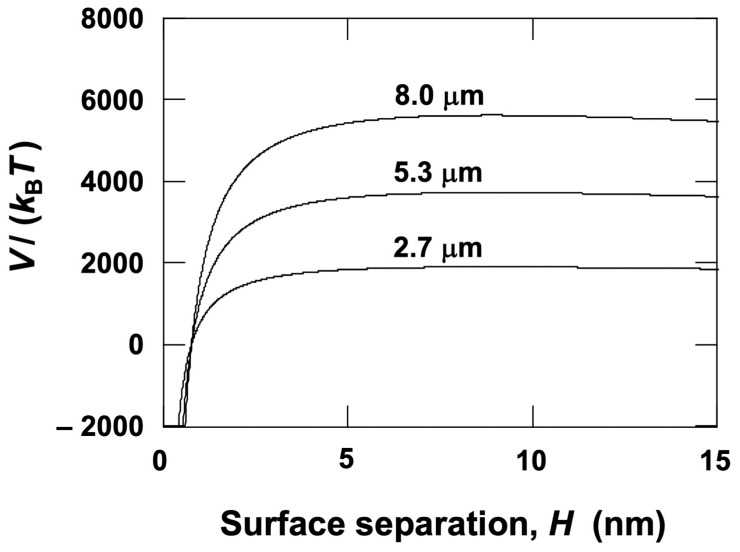
Potential energy curve between PMMA particle surfaces in water at 25 °C. *c* = 1.0 × 10^–5^  mol/L, *L*_D_ = 96 nm, *A*_H_ = 6.3 × 10^–20^  J. *Ψ*_0_ was replaced with the *ζ*-potential (∣*ζ*∣ = 40 mV).

Figure 1 shows the resulting potential energy curves. The vertical axis represents the total potential energy divided by the thermal energy, indicating how much larger the interaction energy between particles is compared to thermal motion. A potential barrier of 25 or higher prevents particle aggregation upon collision [1]. The PMMA particles used in this study are predicted to remain individually dispersed in water, leading to independent sedimentation of the particles.

### 3.2. ERS Effect in Deionized Aqueous Dispersions of Mixed-Sized PMMA Particles

**Figure 2:**
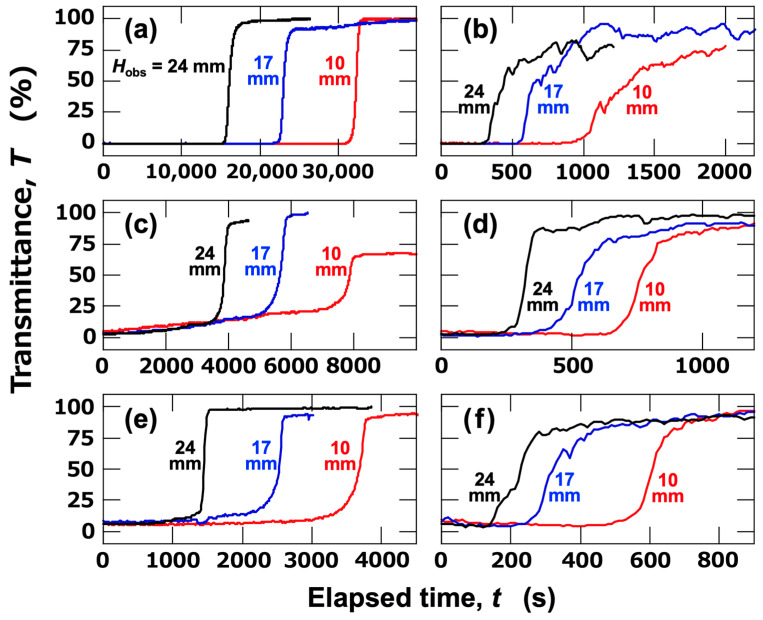
Changes in transmittance of PMMA aqueous dispersions at 25 °C. (**a**,**b**) *d*_PMMA_ = 2.7 μm, (**c**,**d**) 5.3 μm, (**e**,**f**) 8.0 μm. (**a**,**c**,**e**) No electric field; (**b**,**d**,**f**) *E* = 0.5 V/mm DC.

**Figure 3:**
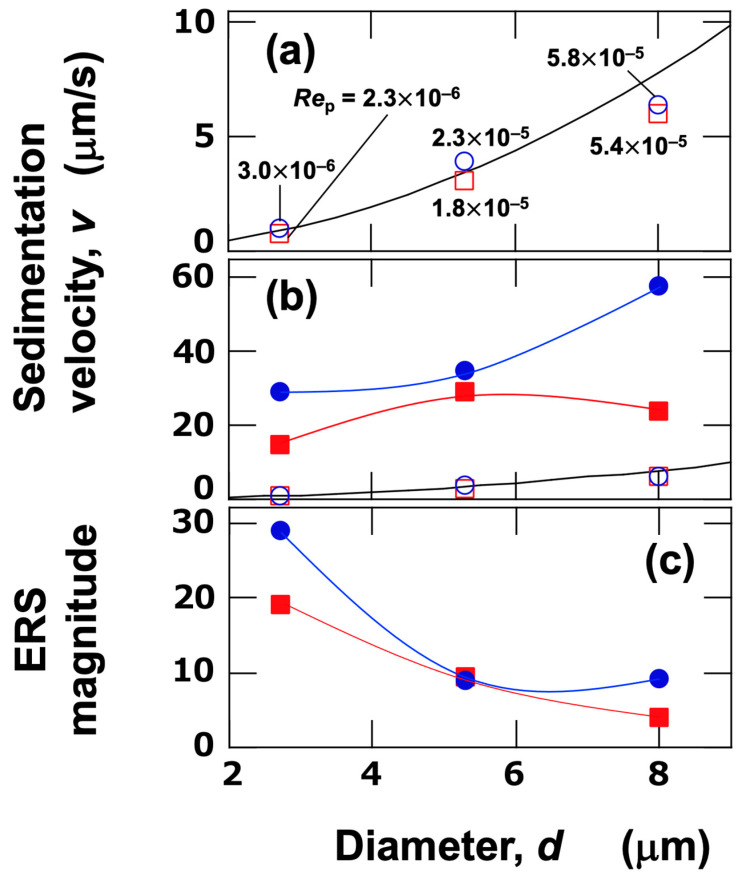
Effect of particle size on sedimentation velocity and the magnitude of the ERS Effect at 25 °C. (**a**) Sedimentation velocity under no electric field. Solid line: theoretical values based on Stokes’ approximation; numbers: particle Reynolds number (*Re*_p_). (**b**) Sedimentation velocity under an electric field (*E* = 0.5 V/mm DC), with comparative data from (**a**). (**c**) The magnitude of the ERS effect. Blue circles: upper region (*H*_obs_ = 17–24 mm); red squares: lower region (*H*_obs_ = 10–17 mm). Open symbols represent data under no electric field, and closed symbols represent data under an electric field.

The ERS effect in aqueous dispersions of single-sized PMMA particles was first investigated. In the dispersions of each particle size, the transmittance exhibited a sudden change at a certain point under no electric field (Figure 2a,c,e). The abrupt increase in transmittance indicates that the interface between the water phase and the colloid-rich phase is sharp. As the interface descended over time and passed through the observation height, an increase in transmittance was observed. The abrupt intensity changes were observed sequentially at observation heights, *H*_obs_ = 24 mm, 17 mm, and 10 mm. When an electric field (*E* = 0.5 V/mm DC) was applied, a significant increase in intensity was observed at a very early stage. This increase in intensity was considerably more gradual than that under no electric field and tended to exhibit small fluctuations. Assuming that flocs formed under the electric field, it can be inferred that the flocs were highly polydisperse in size. The average sedimentation velocity of particles between observation points was calculated based on the time at which the transmittance reached 50% and the distance between observation points (Figure 3). Three observation heights were set in the observation cell. The sedimentation velocity of particles was calculated for the lower region (*H*_obs_ = 10–17 mm) and the upper region (*H*_obs_ = 17–24 mm). The sedimentation velocity of particles under no electric field (Figure 3a) increased with increasing particle size. The average sedimentation velocity in the upper region tended to be slightly larger than that in the lower region. This was likely due to stronger particle-particle interactions in the more concentrated lower region. As mentioned earlier, the thickness of the electric double layer was 96 nm, which is significantly thinner than the size of the colloidal particles. When compared with the theoretical values (solid lines) calculated under the assumption that Stokes’ approximation holds, good agreement was observed for *d*_PMMA_ = 2.7 μm and 5.3 μm, while deviations occurred for *d*_PMMA_ = 8.0 μm. The particle Reynolds number (*Re*_p_) can be expressed as follows [14]:

(4) Rep=ρwvdη

where *ρ*_w_ is the density of water, *v* is the particle sedimentation velocity, *d* is the particle diameter, and *η* is the viscosity of water (0.00089 Pa⋅s at 25 °C). The *Re*_p_ values are shown in Figure 3a. All Reynolds numbers are sufficiently small, indicating that the Stokes approximation holds. Additionally, the Péclet number is expressed by the following equation [15]:

(5) $$\mathrm{Pe}=\frac{\pi d^{4}\Delta \rho g}{12k_{B}T}$$

where Δ*ρ* is the density difference between the particles and water. The Péclet numbers were approximately 6.6, 98, and 511 for *d*_PMMA_ = 2.7, 5.3, and 8.0 µm, respectively. For *d*_PMMA_ = 8.0 µm PMMA particles, the larger Péclet number suggests that they are more susceptible to the influence of convection within the cell, which likely caused the deviation from the Stokes approximation. This is further supported by the fact that the sedimentation velocity in the lower region is slightly smaller than in the upper region. These results, combined with the DLVO potential curves in Figure 1, indicate that under no electric field, the interference with surrounding particles could be neglected, and individual particles sedimented independently without forming flocs. On the other hand, when an electric field was applied, the sedimentation velocity significantly increased (Figure 3b). Additionally, the difference in average sedimentation velocities between the upper and lower regions became more pronounced. Similar to the case without an electric field, the average sedimentation velocity tended to increase with particle size. The sedimentation velocity of flocs formed under the electric field is likely dependent on their fractal dimension [16], and the shape of the flocs during sedimentation may change in a complex manner due to electroosmotic flow and multiparticle interactions. The fractal dimension is a numerical indicator of the “complexity” of objects or structures that exhibit self-similarity. Specifically, the fractal dimension of flocs composed of fine particles in water is an important parameter related to the structure and growth process of the flocs. The magnitude of the ERS effect was defined as the ratio of the average sedimentation velocity under the electric field to that without the electric field, as shown in Figure 3c. The magnitude of the ERS effect indicates how many times the sedimentation velocity increases upon the application of an electric field. The magnitude of the ERS effect decreased with increasing particle size. One possible reason is the lower number density of larger particles compared to smaller ones. This suggests that the ERS effect diminishes or disappears when the distance between particles exceeds a certain threshold.

**Figure 4:**
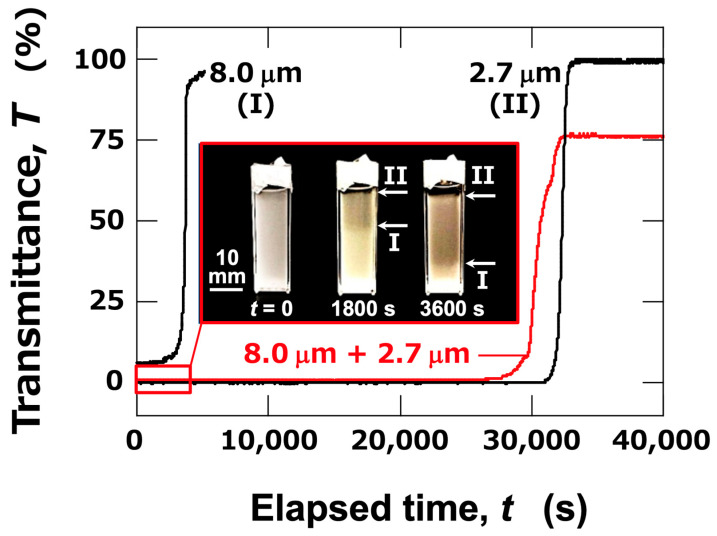
Time-dependent transmittance of mixed aqueous dispersions of large (*d*_PMMA_ = 8.0 μm) and small (*d*_PMMA_ = 2.7 μm) particles (red line) at 25 °C. For reference, data for single-component dispersions of large and small particles are also shown (black lines). Interfaces due to sedimentation of large particles (I) and small particles (II) are labeled. *ϕ*_total_ = 5.0 × 10^–4^ (volume fractions of large and small particles were each 2.5 × 10^–4^). *H*_obs_ = 10 mm. Images show side views of the transparent cell containing mixed dispersions (*t* = 0–3600 s).

Next, the ERS effect in aqueous dispersions of mixed-sized PMMA particles was investigated. In the mixed aqueous dispersions of large particles (*d*_PMMA_ = 8.0 μm) and small particles (*d*_PMMA_ = 2.7 μm), the transmittance under no electric field exhibited a sharp increase around the elapsed time, t = 30,000 s (Figure 4). This timing closely corresponds to the increase observed in the aqueous dispersion of large particles, suggesting that the change in transmittance was primarily due to the sedimentation of large particles. The cell used for the transmittance measurement was photographed from the side (images within Figure 4). Since the dispersion was quite turbid, the brightness, contrast, and sharpness of the images were adjusted from the originals for better visibility. From the images, it was evident that the particles were uniformly dispersed throughout the dispersion immediately after resting (*t* = 0). At *t* = 1800 s, two interfaces (I and II) were relatively clearly observed with the naked eye. Interface (I) corresponded to the sedimentation of large particles, while interface (II) was due to the sedimentation of small particles. As time progressed (*t* = 3600 s), the two interfaces were seen to descend independently. Although the two interfaces could be observed with the naked eye, the transmittance changes only reflect interface (II), which is associated with the sedimentation of small particles. This was likely due to the high particle concentration in the dispersion, which caused significant turbidity, obscuring interface (I) in the transmittance measurements.

**Figure 5:**
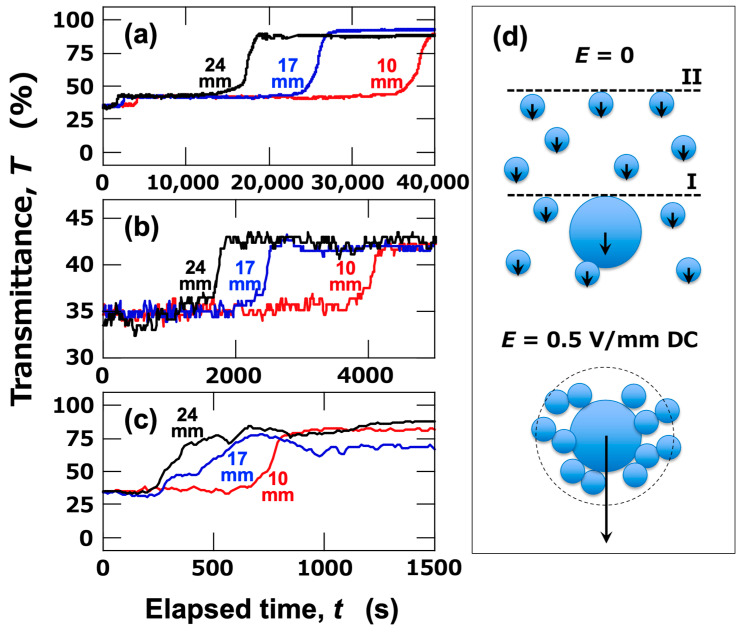
Time-dependent transmittance of mixed aqueous dispersions of large (*d*_PMMA_ = 8.0 μm) and small (*d*_PMMA_ = 2.7 μm) particles at 25 °C. *ϕ*_total_ = 8.0 × 10^–5^ (volume fractions of large and small particles were each 4.0 × 10^–5^). (**a**) No electric field; (**b**) enlarged view of the initial stage (*t* = 0–5000 s) under no electric field; (**c**) *E* = 0.5 V/mm DC; (**d**) schematic diagrams of sedimentation behavior under no electric field and an electric field. Black lines: *H*_obs_ = 24 mm; blue lines: *H*_obs_ = 17 mm; red lines: *H*_obs_ = 10 mm.

To address this, the volume fraction of the mixed aqueous dispersion was diluted to *ϕ*_total_ = 8.0 × 10^–5^ (Figure 5). Under no electric field (Figure 5a), two distinct changes in transmittance were observed: a small change at an early stage and a sharp change occurring later. These corresponded to the passage of interface (I) and interface (II) observed in Figure 4, respectively. Because the intensity change associated with the interface (I) was small, an enlarged view is shown in Figure 5b. The timing of the intensity change varied across observation points. Assuming a constant sedimentation velocity between observation points, the descent velocities of each interface were calculated (Table 1). Interface (I) showed a descent velocity similar to that of the larger particles, while interface (II) exhibited a velocity close to that of the smaller particles (see Figure 3a). This confirmed that the large and small particles sedimented independently without forming flocs upon contact. In contrast, when an electric field was applied (Figure 5c), a significant increase in intensity was observed at a very early stage, occurring only once throughout the entire period. The descent velocity of the interface, in this case, was close to the sedimentation velocity observed in the large-particle dispersion under an electric field (see Figure 3b). The fact that the transmittance increase occurred in a single step indicates that, under the electric field, small particles associated with large particles form flocs (co-flocs) and rapidly sediment together. This result revealed that when large particles are present in the system, smaller particles can form co-flocs with the large particles, enabling rapid sedimentation under the electric field. Since the number density of small particles is much higher than that of large particles, it is suggested that under the electric field, both large and small particles formed flocs and sedimented together, as depicted in Figure 5d. The dotted circle around the flocs reflects the Stokes diameter determined from the sedimentation velocity.

### 3.3. Effect of Rotational Oscillation with a Vertical Axis on the ERS Effect

**Figure 6:**
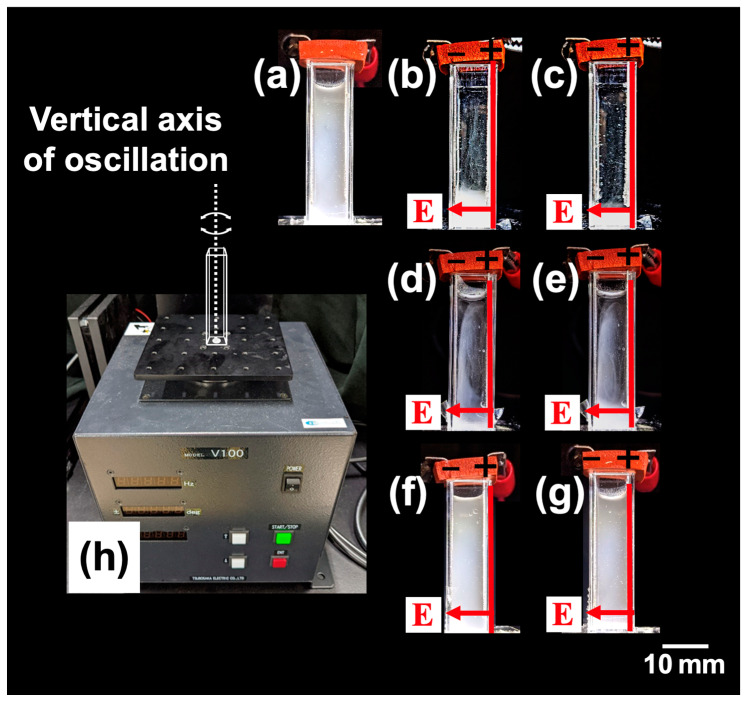
The visual appearance of PMMA aqueous dispersions on a horizontally oscillating platform at 25 °C. *A*_pp_ = 1.6 deg. (**a**) No electric field immediately after resting; (**b**–**g**) *E* = 0.5 V/mm DC; (**b**,**d**,**f**) *t* = 900 s; (**c**,**e**,**g**) *t* = 1800 s. (**b**,**c**) *f* = 1.0 Hz; (**d**,**e**) *f* = 6.0 Hz; (**f**,**g**) *f* = 20 Hz. (**h**) Vibration apparatus with a horizontally oscillating platform with a vertical rotational axis.

**Figure 7:**
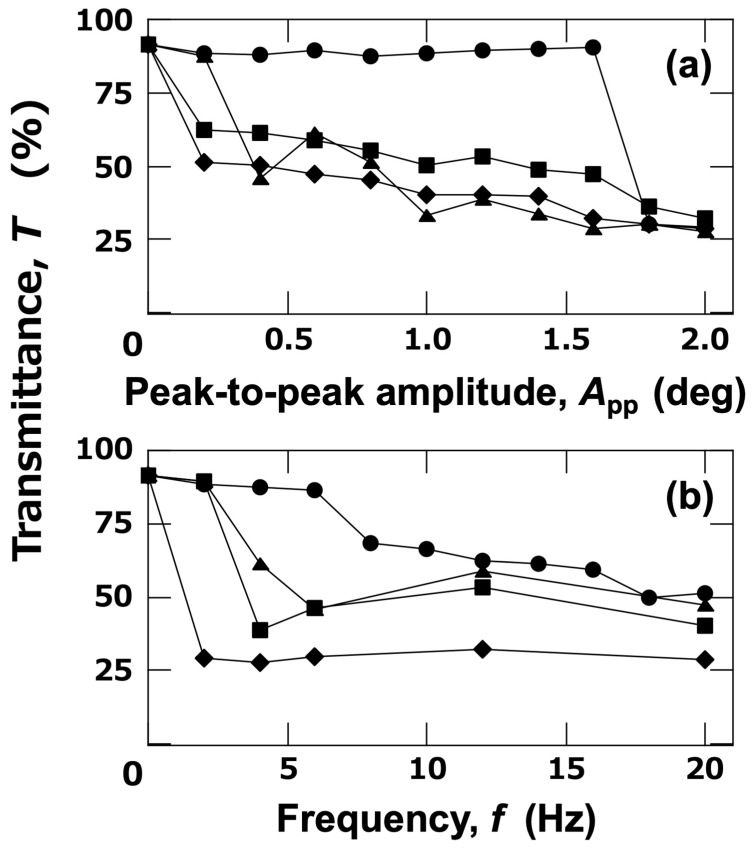
Effect of (**a**) *A*_pp_ and (**b**) *f* on transmittance of PMMA aqueous dispersions at 25 °C. Central point between electrodes; *H*_obs_ = 27 mm. (**a**) ●: *f* = 2.0 Hz; ▲: 4.0 Hz; ■: 12 Hz; ◆: 20 Hz. (**b**) ●: *A*_pp_ = 0.2 deg; ▲: 0.6 deg; ■: 1.2 deg; ◆: 2.0 deg. The solid lines connecting the data points are for better visibility.

The influence of vibration on the dispersion state of PMMA particles was investigated. The ERS effect (*E* = 0.5 V/mm DC) was observed under conditions where the vibration amplitude, *A*_pp_, was fixed at a very small value of 1.6 deg (Figure 6a–g). The horizontal platform of the vibration apparatus (Figure 6h)) oscillated with the vertical direction as its axis of rotation. The behavior of the system up to *t* = 1800 s is shown. Under no electric field and stationary conditions, PMMA particles showed almost no sedimentation by *t* = 1800 s. When an electric field (*E* = 0.5 V/mm DC) was applied, nearly all PMMA particles settled near the bottom of the cell by *t* = 900 s at a vibration frequency of *f* = 1.0 Hz, which was consistent with the ERS effect observed under stationary conditions. At *f* = 6.0 Hz, some PMMA particles sedimented, but others appeared to have their sedimentation inhibited. This inhibited region appeared as vertical bands, suggesting that the flocculation of PMMA particles was suppressed in regions influenced by vibration. At *f* = 20 Hz, sedimentation of PMMA particles was hardly observed even at *t* = 1800 s, indicating that flocculation of the particles was entirely suppressed. The transmittance of PMMA aqueous dispersions after 1800 s under vibrations controlled by *A*_pp_ and *f* was also investigated (Figure 7). Transmittance tended to decrease with increasing *A*_pp_ (Figure 7a). At higher *f*, a decrease in transmittance occurred even at smaller *A*_pp_. The same data plotted with *f* on the horizontal axis is shown in Figure 7b. Transmittance decreased as *f* increased, indicating that even slight vibrations could suppress the ERS effect.

### 3.4. Effect of pH on the ERS Effect in Aqueous Dispersions of Silica Particles

**Figure 8:**
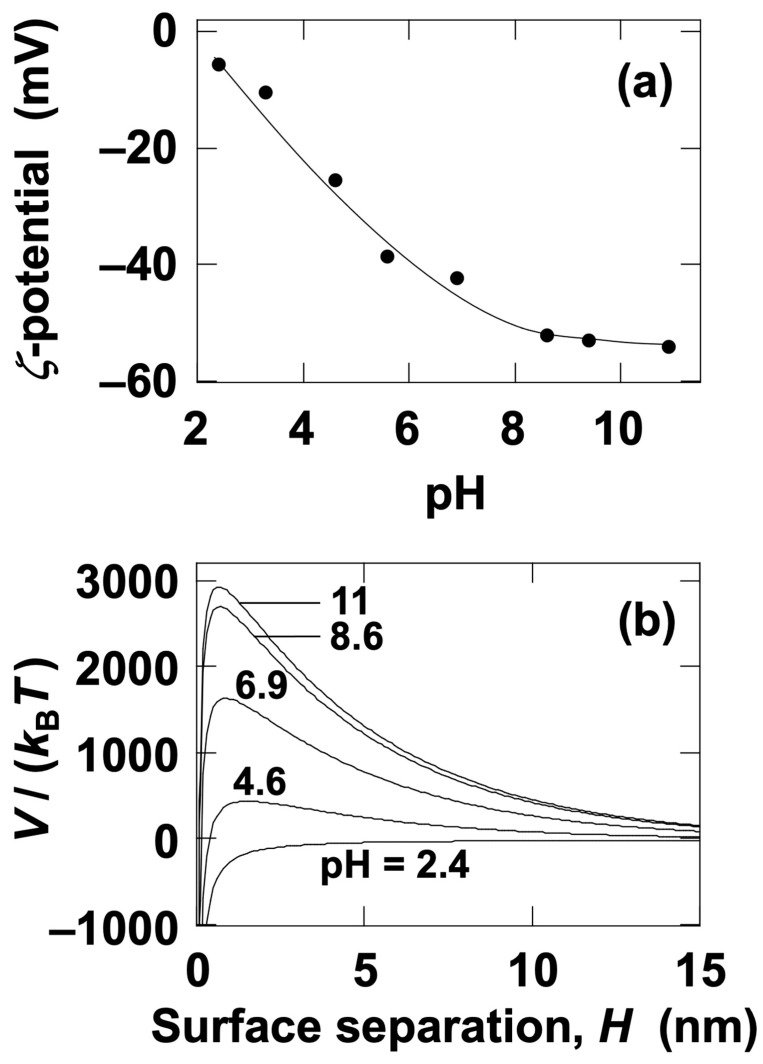
Effect of pH on (**a**) *ζ*-potential and (**b**) DLVO potential energy curves for silica particles in water at 25 °C. *c* = 4.0 × 10^–3^ mol/L; *L*_D_ = 4.8 nm; *A*_H_ = 3.0 × 10^–20^ J. *Ψ*_0_ was replaced with the *ζ*-potential. The solid line in (**a**) is provided to make the changes in *ζ*-potential easier to observe.

The effect of pH on the *ζ*-potential of silica particles in water was investigated (Figure 8a). When pH is simply adjusted, the electrolyte concentration increases as the pH deviates from 7. Since the electrolyte concentration significantly affects the *ζ*-potential, the electrolyte concentration of all sample solutions was unified at *c* = 4.0 × 10^–3^ mol/L using sodium chloride aqueous solutions. The *ζ*-potential exhibited negative values across all pH ranges and tended to increase in absolute magnitude with increasing pH. At *c* = 4.0 × 10^−3^ mol/L, the *L*_D_ value was calculated to be 4.8 nm using Equation (3). To evaluate the dispersion stability of silica particles, DLVO potential curves were plotted using Equation (1) (Figure 8b). It was observed that the barrier of the potential curve increased with pH. Since the maximum barrier values were sufficiently high, particle association due to electrostatic repulsion would not occur even when particles approached each other. At the lowest barrier, corresponding to pH = 2.4, no potential barrier was present. However, the attractive force between particles was extremely weak and could be considered negligible until the surface separation became very small. The center-to-center distance, *L*_cc_, between individual particles in water, assuming an FCC crystal lattice, can be expressed as:

(6) $$L_{\mathrm{cc}}=0.904\;d\;\phi^{–\frac{1}{3}}$$

**Figure 9:**
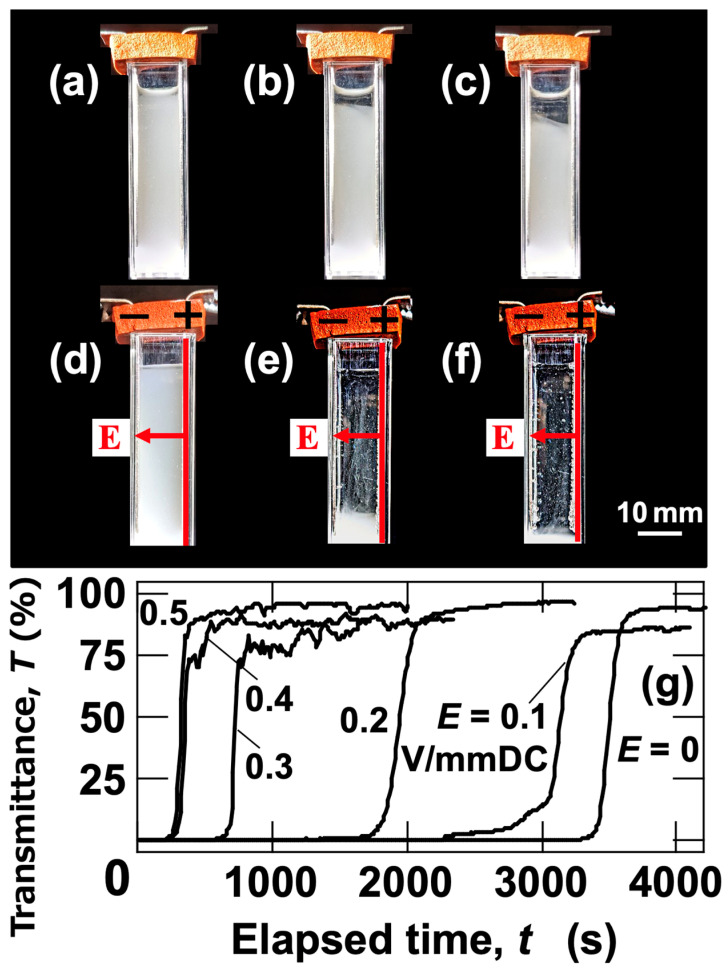
(**a**–**f**) Sedimentation behavior of silica particles and (**g**) transmittance changes under varying electric field strengths at 25 °C. *ϕ*_Si_ = 1.0 × 10^–4^. (**a**) *E* = 0, *t* = 0; (**b**) *E* = 0, *t* = 1800 s; (**c**) *E* = 0, *t* = 3600 s; (**d**) *E* = 0.5 V/mm DC, *t* = 0; (**e**) *E* = 0.5 V/mm DC, *t* = 1800 s; (**f**) *E* = 0.5 V/mm DC, *t* = 3600 s. (**g**) *H*_obs_ = 27 mm.

For the particle volume fraction used in this section (*ϕ* = 1.0 × 10^–4^), *L*_cc_ was calculated to be 48.7 μm, indicating that particles were significantly separated. Therefore, even at pH = 2.4, the likelihood of particle contact and floc formation under no electric field is low. It is also noteworthy that discrepancies between DLVO theory and experimental results for silica particles have been recognized for a long time. Specifically, an additional repulsive force not accounted for in DLVO theory must be considered for silica particles. This additional repulsion is thought to arise from polymer chains on the silica particle surfaces [17]. In summary, even at pH = 2.4, the likelihood of silica particles forming flocs is low. To confirm the manifestation of the ERS effect in aqueous dispersions of silica particles, sedimentation behavior under no electric field and with an applied electric field was observed (Figure 9a–f). Under no electric field, the slow descent of the interface between the water phase and the colloid-rich phase, associated with the sedimentation of silica particles, was observed (Figure 9a–c). Even after *t* = 3600 s, the interface remained in the upper region of the cell. In contrast, with an applied electric field (*E* = 0.5 V/mm DC), almost all the particles appeared to have sedimented to the bottom of the cell within *t* = 1800 s (Figure 9d–f). While the descent of the interface under the electric field was faster than under no electric field, it maintained an almost “horizontal” state, similar to the behavior observed under no electric field. This trend was also observed in the ERS effect of PMMA particle dispersions and other particle dispersions discussed earlier. Transmittance measurements at an observation height of *H*_obs_ = 27 mm were conducted (Figure 9g). Under no electric field, the increase in transmittance was observed at the latest time point, whereas with increasing electric field strength, the increase was observed at earlier time points. The timing of the transmittance increase converged around *E* = 0.5 V/mm DC. These results confirmed that the ERS effect also occurs in aqueous dispersions of silica particles.

**Figure 10:**
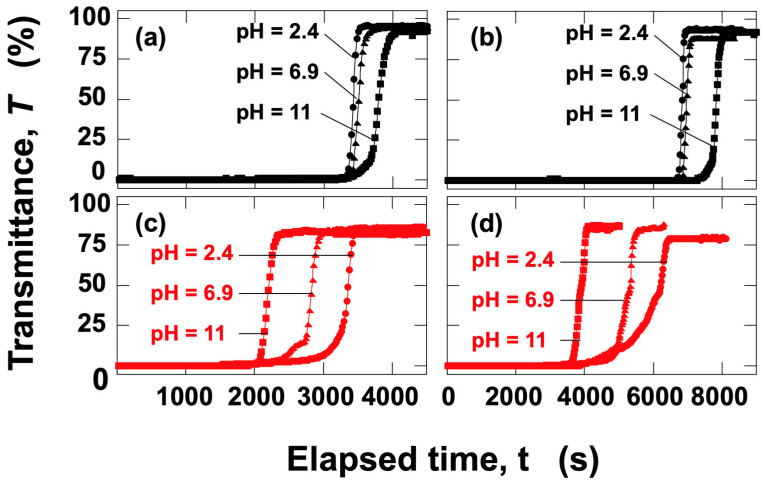
Time-dependent transmittance of silica aqueous dispersions at 25 °C. *ϕ*_Si_ = 1.0 × 10^–4^. (**a**) *E* = 0, *H*_obs_ = 27 mm; (**b**) *E* = 0, *H*_obs_ = 17 mm; (**c**) *E* = 0.1 V/mm DC, *H*_obs_ = 27 mm; (**d**) *E* = 0.1 V/mm DC, *H*_obs_ = 17 mm. Circles: pH = 2.4; triangles: pH = 6.9; squares: pH = 11.

**Figure 11:**
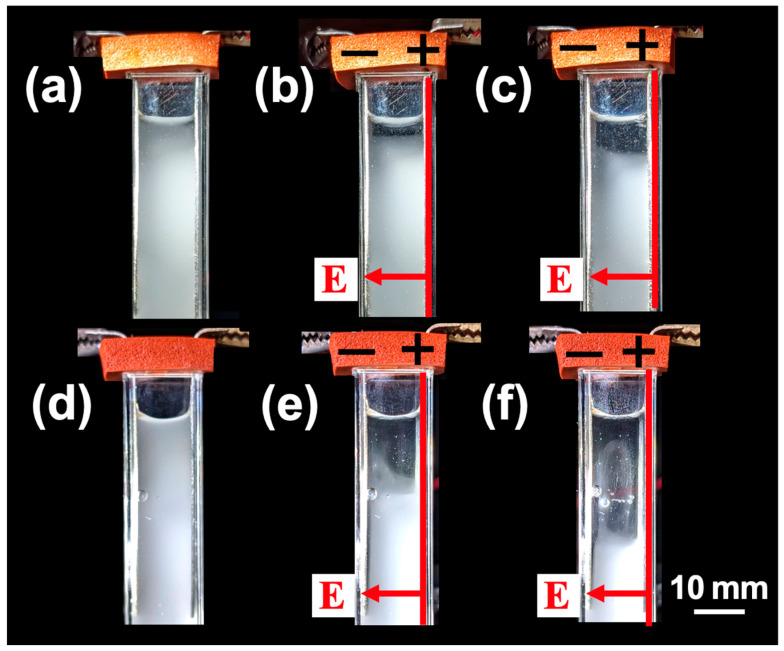
pH-dependent sedimentation behavior of silica particles (*E* = 0.1 V/mm DC) at 25 °C. *ϕ*_Si_ = 1.0×10^–4^. (**a**) pH = 2.4, *t* = 0; (**b**) pH = 2.4, *t* = 1800 s; (**c**) pH = 2.4, *t* = 3600 s; (**d**) pH = 11, *t* = 0; (**e**) pH = 11, *t* = 1800 s; (**f**) pH = 11, *t* = 3600 s.

The time-dependent transmittance of silica particle aqueous dispersions was measured at *H*_obs_ = 27 mm (Figure 10a,c) and *H*_obs_ = 17 mm (Figure 10b,d) for pH = 2.4, 6.9, and 11. Under no electric field (Figure 10a,b), the earliest transmittance increase was observed at pH = 2.4, followed by pH = 6.9, and then pH = 11. This indicates that the sedimentation velocity of individual silica particles was highest at pH = 2.4, followed by pH = 6.9, and lowest at pH = 11. Considering that the particles are sedimented individually, the pH-dependent sedimentation velocity is likely attributable to the influence of the electric double layer surrounding the silica particles. At higher pH levels, the absolute value of the *ζ*-potential is larger, suggesting that the hydrodynamic size of the particles increases with pH. As the hydrodynamic size increases, the fluid resistance during sedimentation also increases, resulting in a corresponding reduction in the terminal sedimentation velocity. Silica particles are more resistant to pH changes compared to PMMA particles; however, under alkaline conditions, particularly at pH = 11, there is a possibility that the silica particles slightly dissolve [18], reducing their particle size within the experimental timeframe of up to approximately 3 h. Despite this potential dissolution of the surface layer, it is remarkable that the silica dispersions exhibited the highest sedimentation velocity under the application of an electric field during this period. In contrast, under an applied electric field (Figure 10c,d), the earliest transmittance increase was observed at pH = 11, followed by pH = 6.9, and lastly, pH = 2.4, a trend opposite to that observed under no electric field. The significant increase in sedimentation velocity under the electric field strongly suggests that silica particles formed flocs. The pH dependence of the sedimentation velocity of silica particles under the electric field was observed (Figure 11). The descent velocity of the interface was clearly the fastest at pH = 11. At pH = 2.4, the interface remained almost horizontal, whereas at pH = 11, the interface appeared more disturbed. At pH = 11, the development of the electric double layer may have induced more complex interfacial electrokinetic phenomena.

**Figure 12:**
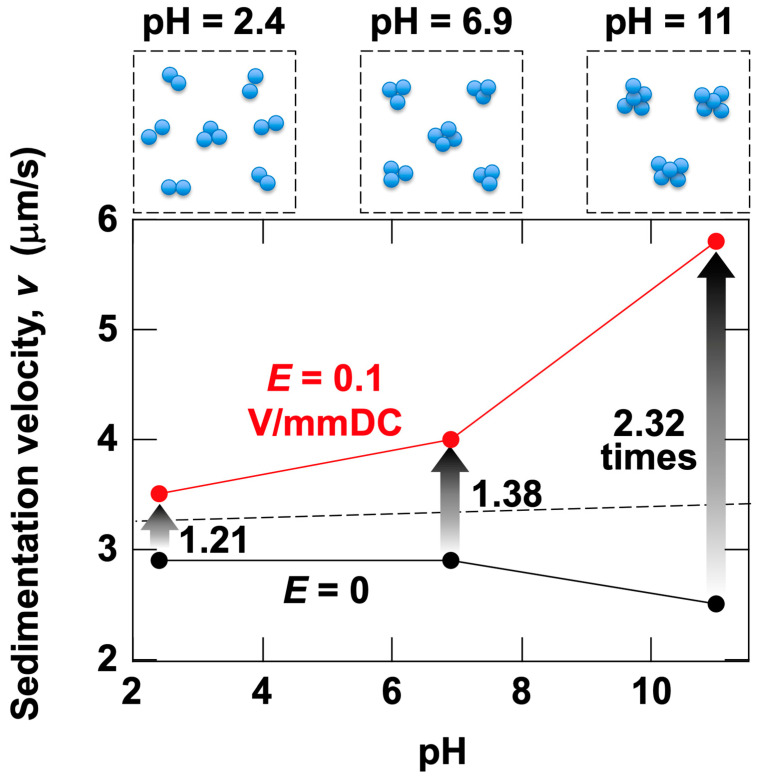
Effect of pH on sedimentation velocity of silica particles in water at 25 °C. *ϕ*_Si_ = 1.0 × 10^–4^. The central point between electrodes. Calculated from the time difference at which transmittance reached 50% at *H*_obs_ = 27 mm and 17 mm. Black circles: *E* = 0; red circles: *E* = 0.1 V/mm DC; dashed line: sedimentation velocity under no electric field calculated using Stokes’ law. The solid lines connecting the data points are for better visibility. The arrows indicate the rate of increase in sedimentation velocity. Schematic diagrams at the top represent aggregates at each pH, with particle number density significantly exaggerated for clarity.

The sedimentation velocity of silica particles was calculated from the time-dependent changes in transmittance (Figure 12). This velocity represents the average sedimentation velocity between observation heights *H*_obs_ = 27 mm and *H*_obs_ = 17 mm, calculated based on the time difference when transmittance reached 50% at each height. Assuming that flocs form under the electric field and that particles are in complete contact, with flocs behaving as single spheres during sedimentation, this can be visualized schematically, as shown at the top of Figure 12. However, the number density of flocs in the schematic is significantly higher than the actual value. As previously mentioned, for *ϕ* = 1.0 × 10^–4^, the *L*_cc_ between particles is 48.7 μm. Remarkably, this indicates that individual particles are separated by a significant distance. To the best of the author’s knowledge, there have been no direct observations to date of colloidal particles forming flocs under a DC electric field in water. While the influence of the electric double layer between particles in water is typically considered to occur over distances on the order of nanometers, it is possible that the electric field extends its influence over longer distances. Additionally, the ERS effect likely involves multiple interfacial electrokinetic phenomena. Further studies are necessary to elucidate the mechanism underlying the ERS effect.

Reports on the ERS effect, including those from this study, are mostly under horizontal electric fields. In such cases, colloidal particles dispersed in water rapidly settle upon the application of a low electric field that does not cause electrolysis [2]. This phenomenon is observed regardless of particle size or shape. In this study, it was demonstrated for the first time that particles of different sizes form flocs together. Additionally, the findings support the conventional understanding that the presence of the electric double layer plays a crucial role in the manifestation of the ERS effect. Furthermore, it was confirmed that vibrations suppress the ERS effect, revealing that the flocs are highly fragile. The ERS effect is significantly influenced by electrophoresis and liquid convection of the dispersion when the direction of the electric field is changed to vertical [3]. Systematic investigations on the ERS effect under varying electric field directions are currently underway, and new findings are expected to be published in the near future.

## 4. Conclusions

When an electric field is applied to and removed from an aqueous colloidal dispersion, the sedimentation velocity of colloidal particles increases reversibly, a phenomenon known as the Electrically Induced Rapid Separation (ERS) effect. This study focused on mixed aqueous dispersions of colloidal particles with different sizes. Under no electric field, it was confirmed that particles were sedimented individually. In contrast, under an electric field, it was found that large and small particles together formed flocs (co-flocs). Since larger floc sizes result in higher sedimentation velocities, systems containing larger particle groups are highly likely to exhibit increased sedimentation velocities for smaller particle groups as well. Small vibrational deformations were found to inhibit the ERS effect. It was suggested that particles forming flocs under an electric field were not in complete contact. Under conditions of a unified electrolyte concentration, the ERS effect showed pH dependency, with larger absolute *ζ*-potential values leading to a more pronounced ERS effect. This indicates that the presence of the electric double layer plays a crucial role in the manifestation of the ERS effect. Previous observations have revealed the formation of horizontal interfaces accompanying particle sedimentation under an electric field, the significant influence of the electric field direction on the ERS effect, and, under certain conditions, the occurrence of convection. These findings suggest that the ERS effect is not solely a matter of particle-particle interactions but involves complex phenomena, including particle electrophoresis, electroosmotic flow of water, and other interfacial electrokinetic phenomena. Floc formation in aqueous colloidal dispersions may also influence the patterns formed during the drying of liquid droplets.

## Figures and Tables

**Table 1:** Sedimentation velocities of boundary interfaces in mixed system aqueous dispersions of large particles (*d*_PMMA_ = 8.0 μm) and small particles (*d*_PMMA_ = 2.7 μm) at 25 °C.

	Sedimentation Velocity (µm/s)		
*H*_obs_ (mm)	*E* = 0 (Ⅰ)	*E* = 0 (Ⅱ)	*E* = 0.5 (V/mm)
17–24	8.5	0.81	47
10–17	4.2	0.57	23

## Data Availability

Data are contained within the article.
